# Evaluation of the Effect of Different Light-Curing Protocols on the Microhardness of Contemporary Bulk-Fill Resin Composites

**DOI:** 10.3390/polym17212889

**Published:** 2025-10-29

**Authors:** Selma Jakupović, Nedim Pervan, Damir Duratbegović, Vedran Jakupović, Enis Muratović, Sedin Kobašlija

**Affiliations:** 1 Department of Restorative Dentistry and Endodontics, Faculty of Dentistry with Dental Clinical Center, University of Sarajevo, 71000 Sarajevo, Bosnia and Herzegovina; jakupovic_selma@yahoo.com; 2 Department of Mechanical Design, Faculty of Mechanical Engineering, University of Sarajevo, 71000 Sarajevo, Bosnia and Herzegovina; muratovic@mef.unsa.ba; 3 Department of Pediatric and Preventive Dentistry, Faculty of Dentistry with Dental Clinical Center, University of Sarajevo, 71000 Sarajevo, Bosnia and Herzegovina; damirduratbegovic@gmail.com (D.D.); skobaslija@sf.unsa.ba (S.K.); 4 Department of Community Healthcare, Faculty of Health Studies, University of Sarajevo, 71000 Sarajevo, Bosnia and Herzegovina; vedran.jakupovic@fzs.unsa.ba

**Keywords:** resin composites, microhardness, bulk-fill composites, high-intensity curing, filler content

## Abstract

Background: The study investigates newly developed composite materials with advanced filler technology and modified resin matrices, designed to enhance esthetic quality, clinical efficiency, and mechanical properties. This study evaluated the effect of two light-curing protocols—a conventional low-voltage (LV) protocol (10 s at 1200 mW/cm^2^) and a high-voltage (HV) protocol (3 s at 3000 mW/cm^2^)—on the microhardness (MH), bottom/top MH ratio, and the correlation between filler content (wt%, vol%) and MH of bulk-fill resin-based composites (RBCs). Four RBCs were tested: Tetric PlusFill (TPF), Tetric Plus Flow(TPFW), Tetric PowerFill (PFL), and Tetric PowerFlow (PFW). Materials and Methods: Samples were fabricated in the laboratory using specially designed cylindrical molds (diameter = 8 mm, height = 4 mm). Initial MH was measured on the top and bottom surfaces of composite specimens 24 h after light curing using a digital microhardness tester (QNESS 60 M EVO, ATM Qness GmbH, Mammelzen, Germany). The correlation between the filler content (wt%, vol%) and the MH of the RBCs was tested. For the calculation of depth-dependent curing effectiveness, the bottom/top ratio for initial MH was used. Conclusions: The MH of bulk-fill RBCs was found to be influenced by both material composition and the applied light-curing protocol. An increase in filler content resulted in higher MH values under both protocols, with the filler volume fraction exhibiting a stronger correlation than the weight fraction. While both flowable and sculptable Tetric Plus composites exhibited higher MH values under the HV protocol, Tetric Power composites demonstrated greater initial hardness under LV protocol. The flowable composite PFW showed the most pronounced reduction in MH under HV curing. The bottom/top MH ratio exceeded 80% in all tested materials, confirming adequate polymerization throughout the composite layers.

## 1. Introduction

Dental resin composites represent one of the most commonly used restorative materials in contemporary dentistry, primarily because of their excellent esthetic qualities, reliable adhesive properties, and superior mechanical performance compared to earlier generations. Their composition typically includes a polymerizable resin matrix, inorganic filler particles, and a silane coupling agent, which together provide mechanical strength, wear resistance, and the ability to achieve a high surface polish [1]. In addition, small amounts of pigments are incorporated to reproduce natural tooth shades, thereby enhancing the esthetic integration of restorations. Furthermore, photoinitiators are included to trigger and control the polymerization reaction under visible light, which is crucial for achieving adequate degree of conversion, mechanical stability, and clinical longevity [2,3]. Advances in filler technology and photoinitiator systems over recent decades have significantly improved the depth of cure, reduced polymerization shrinkage, and increased the long-term clinical stability of these materials [3,4]. As a result, resin composites are now regarded as the restorative material of choice for both anterior and posterior teeth, offering a well-balanced combination of esthetics, functionality and durability.

Bulk-fill composites represent a class of restorative materials designed to be applied in increments of 4–5 mm, unlike conventional composites that require layering in 2 mm thicknesses. This simplified application reduces chair time and minimizes the risk of incorporating voids between layers [5]. Bulk-fill composites are formulated to enhance light transmission through deeper increments. Modification of filler content, particle size distribution, and resin opacity, combined with the use of advanced photoinitiators, allows for sufficient depth of cure without compromising mechanical strength. Studies demonstrate that both high- and low-viscosity bulk-fills can achieve good marginal integrity, although viscosity, placement technique, and curing protocol strongly influence performance [6,7].

Contemporary bulk-fill composites, such as Tetric Plus (Fill/Flow) and Tetric Power (Fill/Flow), which are examined in this study, represent recent advancements in composite technology. All four composites were designed for polymerization using both the LV and HV protocols. Their formulations incorporate innovations such as the germanium-based photoinitiator Ivocerin, enhancing light absorption and polymerization efficiency, together with an optimized filler system that improves depth of cure, mechanical properties, and esthetic stability. Studies indicate that Tetric PowerFill performs reliably under both conventional and fast curing protocols, maintaining adequate degree of conversion and mechanical performance even under high irradiance and short exposure times [8,9].

Tetric Plus is a newly introduced universal composite from Ivoclar, designed to enhance both efficiency and aesthetics in direct restorations. It can be applied in layers up to 4 mm and is available in two viscosities: sculptable and flowable [8].

Currently, there are no peer-reviewed clinical publications available on Tetric Plus. The type of filler technology plays a crucial role when investigating the microhardness of composite materials. Tetric Plus utilizes a next-generation 4-filler system, designed to optimize handling, esthetics, depth of cure, and mechanical properties. Nano Ytterbium Trifluoride (nano-YbF3) provides enamel-like radiopacity (≥200% Al) and contributes to the material’s pronounced translucency shift. Very small particle size ensures that light transmission is not compromised, allowing excellent depth of cure while maintaining flowable or sculptable handling properties depending on application pressure. Glass Filler is the core filler responsible for mechanical strength and flexural modulus. Its refractive index is matched to the monomer matrix, supporting translucency shift and high polishability (particle size ~0.7 μm). Optimized Mixed Oxide (SiO_2_/ZrO_2_) consists of small spherical primary particles that form clusters, enhancing surface qualities, allowing for quick polishability, and providing a smooth consistency. Spherical IFP (In-Flight Polymerized) Composite Filler contains pre-polymerized spherical particles that improve flow and handling, ensure uniform distribution within the matrix, reduce viscosity, and maintain the translucency shift [8].

The polymerization of resin-based dental composites is achieved through light-curing protocols that activate photoinitiators within the resin matrix by specific wavelengths of light (typically 400–500 nm). This activation initiates a free-radical polymerization process, leading to the formation of a cross-linked polymer network. The degree of conversion (DC) during this process is crucial, as it directly influences the mechanical properties, wear resistance, and biocompatibility of the composite material [10].

Effective curing depends on factors such as composite thickness, opacity, and filler content, as well as the distance and angle of the light source. Traditional protocols often use moderate light intensity over longer exposure times (e.g., 1000 mW/cm^2^ for 10–20 s), while newer high-intensity protocols allow for rapid curing in shorter times (e.g., 3000 mW/cm^2^ for 3 s) [11,12]. This rapid curing method is intended to shorten treatment time and improve clinical efficiency. However, the increased light intensity raises concerns regarding the potential rise in temperature within the dental pulp. Studies have indicated that rapid curing can lead to elevated transdentinal temperatures, which may affect pulp vitality and patient comfort. Miranda et al. (2024) reported that rapid high-intensity light-curing increased transdentinal temperature and cell viability, highlighting the need for careful consideration of curing protocols to mitigate potential risks [13].

Regardless of the protocol, the goal is to achieve a sufficient DC, adequate microhardness, and long-term mechanical stability of the restoration [14]. Both light-curing protocols have their advantages and limitations. The choice between extended exposure and high-intensity exposure should be guided by the specific requirements of the restorative material, the clinical situation, and the capabilities of the light-curing unit.

In bulk-fill composites achieving a high DC is particularly important due to the increased layer thickness, which can reach up to 4 mm [10,15]. Research has demonstrated that appropriate light-curing protocols, including both standard and ultra-fast high-intensity methods, significantly affect DC and MH [10,16,17,18]. For instance, ultra-fast curing with high-intensity light (3 s exposure) can provide satisfactory DC and MH; however, special attention is needed to ensure adequate polymerization at greater depths, as MH typically decreases with increasing distance from the light source [13,19].

Consequently, MH measurements are widely used as an indirect method to assess DC in both conventional and bulk-fill composites, allowing evaluation of the effectiveness of different curing protocols and their impact on the mechanical performance and long-term durability of the material [10]. Higher MH values correlate with higher biocompatibility of composite filling [20,21]

Despite extensive literature on conventional composites, the performance of contemporary materials—incorporating advanced filler technologies and modified resin matrices designed to enhance esthetics, improve micromechanical properties, and enable faster clinical application—remains insufficiently investigated, particularly regarding their response to different light-curing protocols and the resulting surface and depth-dependent microhardness.

The aim of this study was to evaluate the effects of high-intensity (HV) and low-intensity (LV) curing protocols on microhardness (MH) values and the MH bottom–top ratio, as well as to investigate the influence of filler content (wt% and vol%) on MH in contemporary bulk-fill composites.

## 2. Materials and Methods

### 2.1. Tested Materials and Light-Curing Protocols

Four different bulk resin composites were used in this study: Tetric PlusFill, Tetric PlusFlow, Tetric PowerFill, and Tetric PowerFlow (Table 1).

Two types of curing protocols were used. The high-voltage (HV) protocol involved light curing for 3 s at an irradiance of 3000 mW/cm^2^, while the low-voltage (LV) protocol used an irradiance of 1200 mW/cm^2^ for 10 s. An LED curing unit (Bluephase PowerCure, Ivoclar Vivadent, Schaan, Liechtenstein; emission wavelength range: 390–500 nm) was employed in this study. Eight experimental groups were created, consisting of four bulk-fill materials (flowable and sculptable) each cured using both HV and LV protocols (Table 2).

### 2.2. Specimen Preparation

A total of 56 composite specimens (8 mm in diameter and 4 mm in height) were prepared, with seven samples per experimental group. Specimens were fabricated in silicone molds open on both sides. The bottom surface was flattened using a glass plate and Mylar strips [23], while the top surface was covered with Mylar strips and a glass slide [24] to achieve proper condensation of the material and a smooth surface suitable for Vickers microhardness measurements.

After removing excess material, specimens were irradiated only from the top side (previously marked) through the Mylar strip in direct contact with the curing tip. Upon polymerization, the upper surface of each specimen was marked, and all samples were stored in light-proof containers filled with distilled and deionized water at 37 ± 1 °C for 24 h to complete the post-polymerization process.

Subsequently, both the upper and lower surfaces of the specimens were polished using silicon carbide polishers at 8000 rotations per minute. Surface residues were removed by washing and drying the specimens. All samples were prepared at room temperature (21–23 °C) and a relative humidity of 40–60%.

### 2.3. Microhardness Measurement Protocol

Vickers microhardness (VMH) was measured on the top and bottom surfaces of each specimen using a digital microhardness tester (QNESS 60 M EVO, ATM Qness GmbH, Mammelzen, Germany) equipped with a Vickers diamond indenter and a 20× magnification microscope. Diamond-shaped indentations were made in both the central and peripheral regions of the specimen surface under a load of 1 kP (kilopond, equivalent to 1000 g) for 15 s [25,26,27,28].

**Figure 1:**
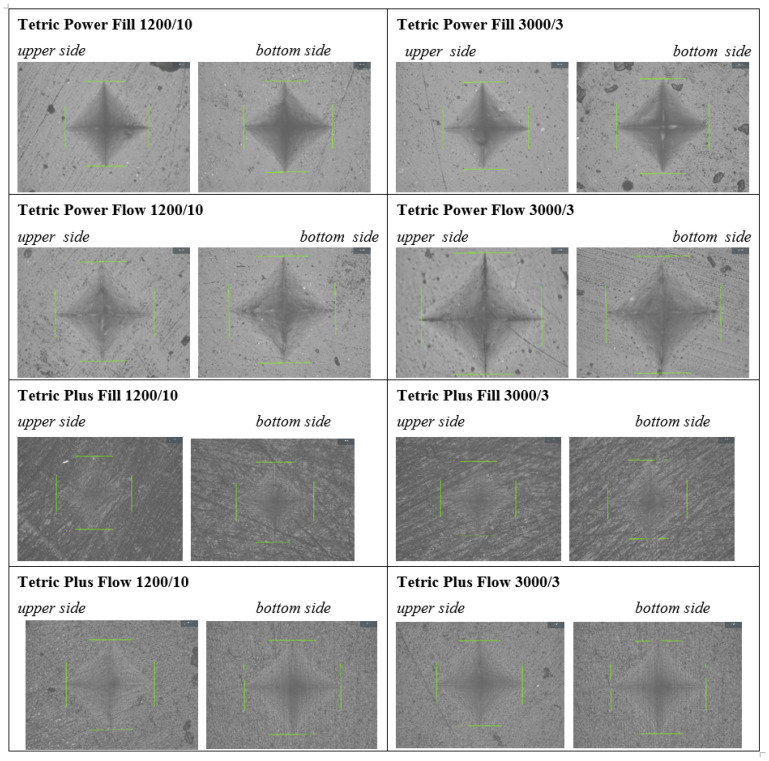
Electromicroscopic images of VMH measurements.

Based on the size of the impressions, the device automatically calculated the VMH values using Equation (1):

(1) $$HV=0.1891\cdot \frac{F}{d^{2}}$$

where *F* is the applied load in Newtons (N) and *d* is the mean diagonal length in millimeters (mm), measured by the integrated electron microscope (Figure 1). Five measurements were taken on each surface of every sample: three in the central region and two in the peripheral region of the discoid specimen. The mean VMH value was then calculated for each surface.

### 2.4. Statistical Analysis

Due to the parametric nature of the aforementioned analysis, the eligibility for their usage has been tested via the Shapiro–Wilk test. Additional checks (such as test of sphericity) were performed for specific tests (i.e., the RMANOVA) to ensure that they may be performed. After these initial checks, the actual tests were performed.

To evaluate the main effects of the restorative material and the polymerization protocol, as well as their interaction on composite MH, the RMANOVA has been used. This method is appropriate for mixed factorial designs, where one factor/variable is defined as between-subjects (type of material/composite) and the other as within-subjects (LV/HV protocols).

The Pearson’s correlation coefficient has been used to test whether a statistically significant relationship exists between the microhardness of the composite, as well as their filler content (i.e., their wt% and vol%, respectively).

## 3. Results

**Figure 2:**
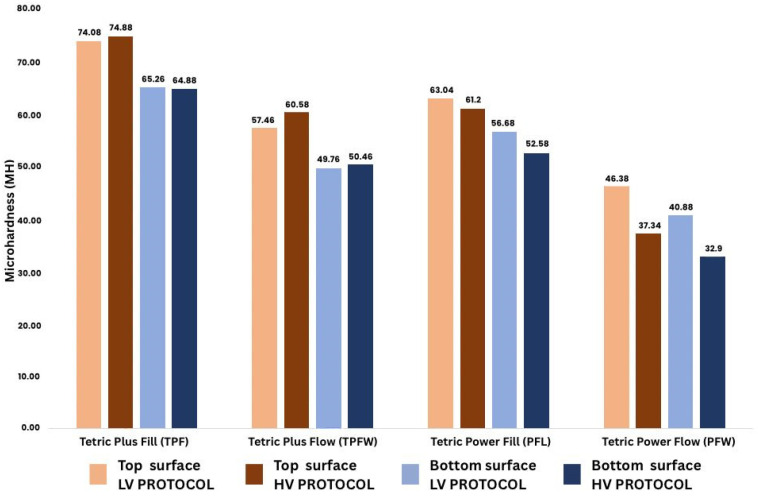
Initial MH of the tested composites.

Initial MH values measured on the top and bottom surfaces of the specimens are presented in Figure 2. For flowable composites, MH ranged from 32.9 to 60.58, while for sculptable composites, values ranged from 52.58 to 74.88 across both curing protocols. The highest MH was observed for the sculptable composite TPF, with values of 74.08 and 74.88 on the top surface and 65.26 and 64.88 on the bottom surface. In contrast, the flowable composite PFW demonstrated the lowest MH, showing a reduction under the high-intensity (HV) curing protocol.

Table 3, shows descriptive initial MH data for the investigated RBCs. In Table 4, statistically significant main effects of the variables material and polymerization can be observed, as well as a significant interaction effect between them. This statistical significance persists regardless of whether MH was measured on the upper or lower surface of the specimen. Thus, there is a statistically significant difference in MH between different composites. Furthermore, a statistically significant difference exists between specimens treated with LV and HV protocol. Partial eta-squared values represent the relative effect sizes of the factors “material,” “curing protocol,” and their interaction on the initial MH of the top and bottom surfaces of the specimens. The variable material exhibits the highest partial eta-squared value, indicating the strongest practical significance, while the other two parameters also demonstrate statistical significance, as reflected by their high partial eta-squared values.

Pearson’s correlation coefficient was used to examine the relationship between MH and composite filler content (wt% and vol%). Correlations were performed using MH values measured on the top surface of specimens polymerized with two different curing protocols [29]. A total of four correlations were analyzed, as presented in Table 5. All four tested correlations were statistically significant, and the correlation coefficients were relatively high. Higher filler content, both by weight and volume, was associated with a corresponding increase in specimen MH, which was observed for both LV and HV protocols. The effect of filler volume showed a stronger correlation.

**Figure 3:**
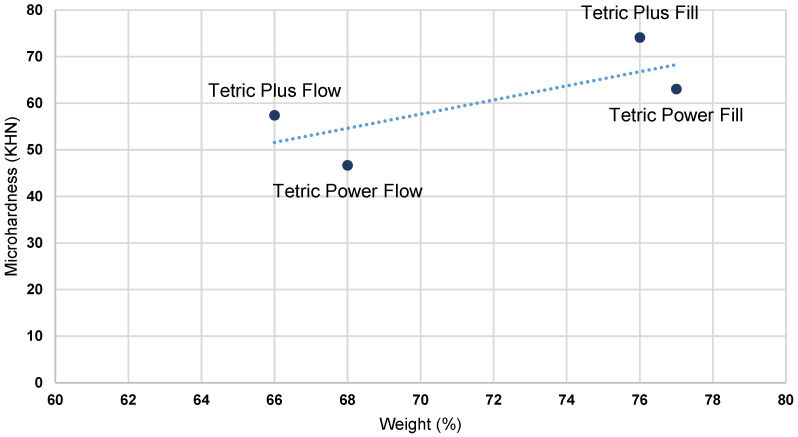
Plots of initial MH measured on the top surface of specimens in relation to filler weight (%)—LV protocol.

**Figure 4:**
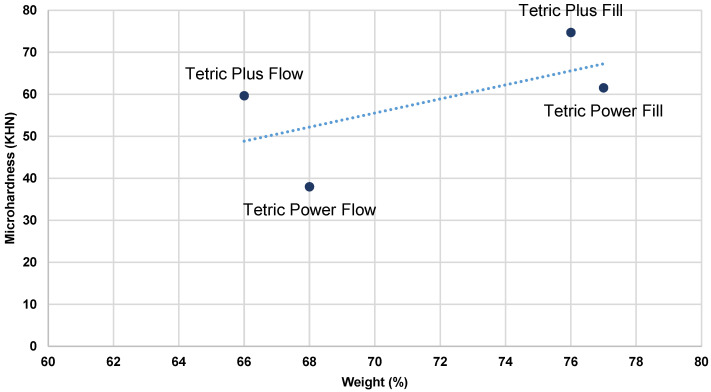
Plots of initial MH measured on the top surface of the specimens in relation to filler weight (%)—HV protocol.

**Figure 5:**
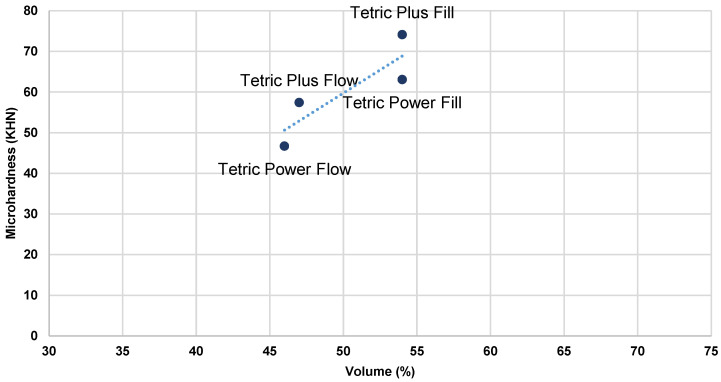
Plots of initial MH measured on the top surface of the specimens in relation to filler volume (%)—LV protocol.

**Figure 6:**
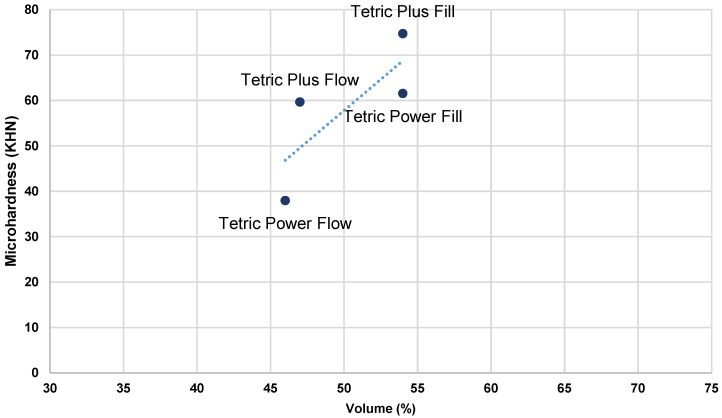
Plots of initial MH measured on the top surface of the specimens in relation to filler volume (%)—HV protocol.

Figure 3, Figure 4, Figure 5 and Figure 6 present the relationship of MH with filler content (wt% and vol %).

**Figure 7:**
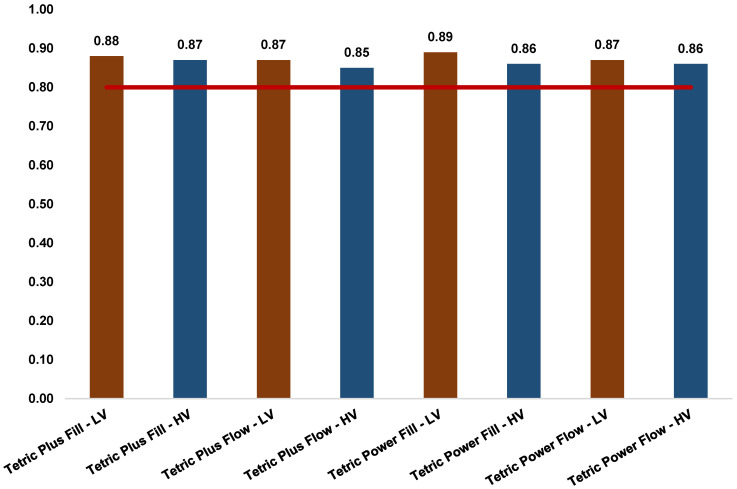
The relationship between the initial MH of the bottom and top surfaces of the speci-mens (bottom/top ratio).

Figure 7 illustrates the relationship between the initial MH of the bottom and top surfaces of the specimens (bottom/top ratio). This ratio should not be lower than 80% (i.e., below 0.8). All four composites meet this criterion, regardless of whether they were polymerized with HV or LV protocol.

## 4. Discussion

Microhardness is recognized as a reliable indirect indicator of the degree of polymerization of resin-based composites, providing valuable insights into their mechanical performance and long-term clinical behavior. During polymerization, the conversion of monomers into a polymer network is never complete and typically reaches up to approximately 75%. The residual unreacted methacrylate groups remaining in the deeper, insufficiently polymerized regions of composite restorations pose potential cytotoxic and genotoxic risks. Moreover, their increased solubility may lead to the formation of voids, marginal degradation, and, consequently, the development of secondary caries, highlighting the importance of evaluating the degree of polymerization [30]. Since the success of composite restorations depends not only on esthetics but also on their durability under masticatory forces, evaluating microhardness offers a practical way to assess the quality of curing and predict the material’s resistance to wear and degradation. Differences in microhardness values can reflect variations in filler composition, resin matrix formulation, curing protocols, and depth of polymerization, all of which are key parameters when analyzing the performance of contemporary bulk-fill composites [31,32]. This study examined the effects of different light-curing protocols (LV and HV) on MH values and the bottom/top MH ratio, as well as the influence of filler content (wt% and vol%) on material performance.

An important step in MH evaluation was the polishing of all samples. All samples were polished on both upper and lower surfaces using silicon carbide polishers at 8000 rpm. Polishing was a crucial step, since surface smoothness can significantly affect the accuracy and reproducibility of MH measurements. A well-polished surface allows uniform contact between the indenter and the composite material, minimizing variability and avoiding stress concentration points that may lead to underestimated MH values. Furthermore, surface polishing has clinical importance, as smoother composite surfaces exhibit improved wear resistance, reduced plaque accumulation, and superior esthetic stability. Therefore, achieving a highly polished surface not only ensured reliable MH testing in this study but also reflected an essential factor for the long-term performance of restorative composites [33,34]. In this study, partial η^2^ values revealed statistically significant effects of both material and curing protocol, as well as a significant interaction effect between these factors on the initial MH of both specimen surfaces. The impact of the curing protocol was slightly lower than that of the material or the interaction effect, indicating that variations in MH are primarily determined by the composite material, while the curing method also contributes to these differences. Similar findings have been reported in other studies [35]. The effect of both the curing protocol and the material was more pronounced on the bottom surface of the specimens.

Recent studies have highlighted the significant impact of light-curing protocols on the microhardness of resin-based composites. Research indicates that curing intensity and duration can influence the microhardness of both bulk-fill and conventional composites, although the filler content often plays a more decisive role [9,27,28]. Other research demonstrated that factors such as curing intensity, exposure time, and light source distance significantly affected MH values and the bottom-to-top hardness ratio, particularly at greater curing depths [15]. Additionally, variations in shade and specimen thickness, combined with beam inhomogeneity, were shown to alter hardness outcomes even under identical curing conditions [36]. Comparisons of different curing modes revealed that certain composites achieved higher hardness values under high-intensity protocols, though the outcome remained material-dependent [37].

Finally, studies evaluating ultrafast protocols (1 s and 3 s) reported that, while these approaches drastically reduced curing time, the bottom surface hardness was often lower compared to conventional curing, unless the composite was specifically optimized for high-intensity light curing [38]. These findings emphasize the importance of optimizing curing parameters and material selection in order to achieve sufficient polymerization throughout the bulk of the composite, thereby ensuring long-term clinical success.

In this study, all four investigated composites were formulated for polymerization using both LV and HV protocols. The highest initial MH values were observed for the sculptable composite TPF under the HV protocol, measuring 74.88 on the top surface and 64.88 on the bottom surface. The PFL composite exhibited lower MH values compared to TPF. A pronounced effect of high-intensity curing on initial MH was noted for the Tetric Plus composites (TPF and TPFW), with all initial MH values being higher under the HV protocol, in contrast to the Tetric Power composites (PFL and PFW) (Figure 2). This is likely a result of the advanced filler technology in these composites, as well as the initiator system. When analyzing the flowable composites, they showed lower initial MH values compared to the sculptable composites. The lowest MH values were recorded for PFW, with 37.34 on the top surface and 32.9 on the bottom surface under the HV protocol. These values were significantly lower than those measured for the other flowable composite TPFW, which exhibited an initial MH of 60.58 under the HV protocol. For TPFW, the HV protocol resulted in higher initial MH on both top and bottom surfaces compared to the LV protocol (Figure 2). The MH values recorded for TPFW are relatively high for a flowable composite and nearly approach the microhardness measured for some sculptable composites. This is likely due to the higher effective filler load in TPFW, which, together with specific thixotropic properties during handling, facilitates its clinical application. The greatest difference in initial MH between the top and bottom surfaces was observed for the flowable composite PFW under the HV protocol. This can be explained by differences in the mobility of reactive species resulting from the varying viscosities of the reaction medium, which have been considered responsible for the commonly reported observation that polymerization effectiveness tends to be more reduced by high-intensity light-curing in flowable than in sculptable composites [3,22,25,39].

As the material composition was identified as the most influential factor for all outcome variables, Pearson’s correlation analysis was conducted to examine the relationship between the top surface MH values and filler content (wt% and vol%) in the tested bulk-fill composites under both HV and LV protocols (Table 5). The results of the analysis revealed statistically significant correlations for all investigated parameters, with r = 0.74 and 0.61 for wt%, and r = 0.87 and 0.78 for vol% under the LV and HV protocols, respectively. A higher filler content, expressed either by weight or by volume, was consistently associated with increased MH values across both curing protocols, with filler volume demonstrating the stronger correlation. Plots of initial MH versus filler content (Figure 3, Figure 4, Figure 5 and Figure 6) showed no substantial deviations from the correlation line. However, slightly lower correlation values were observed under the HV protocol for both vol% and wt%. The weaker correlation associated with the HV protocol may be explained by the material-dependent effects of high-intensity curing on the polymer network structure, which diminished the relative contribution of filler content to the determination of composite MH [35]. This effect was particularly pronounced in the flowable composite PFW.

According to the widely accepted criterion, a bottom-to-top MH ratio exceeding 80% is considered indicative of adequate polymerization throughout the composite layer [40,41,42,43]. The bottom surface of the composites exhibits lower hardness compared to the top surface, which can be attributed to the reduced polymerization resulting from the lower light energy input. In this study, all examined composites demonstrated satisfactory bottom-to-top ratios for both curing protocols, and consequently an adequate degree of conversion, with values ranging from 0.85 to 0.89 (Figure 7). Previous studies have reported that the decrease in MH in the deeper layers of specimens is significantly less pronounced in bulk-fill composites [44]. The observed behavior can be explained by the fact that in uncured state, the examined composites exhibit high translucency, which enables increased light penetration. This property is essential for achieving a high degree of conversion and reliable curing of increments up to 4 mm, as light transmission is more effective through a transparent medium. During polymerization, however, the density of the organic matrix increases while translucency decreases, resulting in a tooth-like esthetic opacity. This shift in translucency is achieved through the precise matching of the refractive indices of the composite’s monomer matrix and inorganic fillers [39,45,46]. Since the refractive index determines the extent to which light waves are refracted and scattered when passing through or interacting with particles in a medium, optimization of the filler content is of central importance for both the optical properties and curing efficiency of resin composites.

## 5. Conclusions

The microhardness (MH) of four contemporary bulk-fill resin-based composites was investigated under two curing protocols differing in light intensity and curing time. The following conclusions can be drawn:

- The MH of RBCs is influenced by both material composition and the applied light-curing protocol.
- Higher filler content, expressed both by weight and volume, was consistently associated with increased MH values under both LV and HV protocols, with filler volume demonstrating the stronger correlation.
- The effect of high-intensity light-curing on micromechanical properties was markedly dependent on material composition. Both flowable and sculptable Tetric Plus composites exhibited higher MH values under the HV protocol on both specimen surfaces, whereas the Tetric Power composites demonstrated greater initial hardness under the LV protocol. The flowable composite PFW showed the most pronounced reduction in MH under the HV protocol.
- Despite these differences, the bottom-to-top MH ratio exceeded 80% for all tested bulk-fill composites, indicating adequate polymerization throughout the composite layers.

The results provide insights for selecting optimal curing protocols for various bulk-fill composites to ensure adequate polymerization and mechanical performance. Clinically, this knowledge can assist dentists in choosing appropriate curing strategies to enhance restoration durability, minimize the risk of incomplete polymerization, and speed up clinical procedures. Future studies could investigate the long-term effects of these protocols on the wear resistance, marginal integrity, and biocompatibility of the tested composites.

## Figures and Tables

**Table 1:** Investigated resin composites [22].

Composite Viscosity	Composite Type	Composite Name	Composition	Filler Content (wt%/vol%)	Manufacturer
Sculptable	Bulk-fill	Tetric PlusFill (TPF) Shade A2	Strontium glass, copolymer, mixed oxide (SiO_2_/ZrO_2_), UDMA, ytterbium trifluoride, Bis-GMA, aromatic-aliphatic UDMA, DCP, Bis-EMA	70/52	Ivoclar Vivadent, Schaan, Liechtenstein
		Tetric PowerFill (TPF) Shade IVA	Barium glass, copolymer, Si-Zr mixed oxide, Bis-GMA, ytterbium trifluoride, Bis-PMA, UDMA, Bis-EMA	77/54	
Flowable	Bulk-fill	Tetric PlusFlow(TPFW) Shade A2	Barium glass, co-polymer, mixed oxide (SiO_2_/ZrO_2_), BPEMA, MOMA, Bis-GMA, Bis-EMA, DCP, UDMA, ytterbium fluoride, AFCT	65/51	
		Tetric PowerFlow(PFW) Shade IVA	Barium glass, Bis-EMA, copolymer, aromatic methacrylate, Bis-GMA, ytterbium trifluoride, DCP	68/46	

**Table 2:** Experimental groups.

Group	Resin Composite Material	Curing Protocol	Sample Size, *n*
1.	Tetric plus Fill	LV-1200 mW/cm^2^/10 s	7
2.	Tetric plus Fill	HV-3000 mW/cm^2^/3 s	7
3.	Tetric power Fill	LV-1200 mW/cm^2^/10 s	7
4.	Tetric power Fill	HV-3000 mW/cm^2^/3 s	7
5.	Tetric plus Flow	LV-1200 mW/cm^2^/10 s	7
6.	Tetric plus Flow	HV-3000 mW/cm^2^/3 s	7
7.	Tetric power Flow	LV-1200 mW/cm^2^/10 s	7
8.	Tetric power Flow	HV-3000 mW/cm^2^/3 s	7

**Table 3:** Initial MH—descriptive statistics.

Material	Average	Standard Deviation	Min.	Max.
Tetric Plus Fill	69.85	4.68	64.66	75.24
Tetric Plus Flow	54.35	4.39	48.36	60.58
Tetric Power Fill	58.38	4.21	52.40	63.38
Tetric Power Flow	39.47	5.15	32.12	47.22

**Table 4:** RMANOVA results (*p*-values and partial eta-squared).

	Top Surface		Bottom Surface	
	*p*	Partial *η*^2^	*p*	Partial *η*^2^
Material	<0.001	0.99	<0.001	1
Curing protocol	<0.001	0.94	<0.001	0.95
Material–curing protocol	<0.001	0.99	<0.001	0.97

**Table 5:** Pearson’s correlation coefficient of composite MH in relation to filler content.

Correlated Variables	Correlation Coefficient, *r*	*p*-Value
wt%–LV protocol	0.74	< 0.001
wt%–HV protocol	0.61	< 0.001
vol%–LV protocol	0.87	< 0.001
vol%–HV protocol	0.78	< 0.001

wt% = weight filler content (%), vol% = volume filler content (%).

## Data Availability

The raw data supporting the conclusions of this article will be made available by the authors on request.
